# Association of hemoglobin level with granulocyte engraftment and red blood cell transfusion by transplant type after hematopoietic stem cell transplantation: a retrospective cohort study

**DOI:** 10.1080/07853890.2026.2729599

**Published:** 2026-09-07

**Authors:** Junqing Wang, Jiaping Wang, Qiming Ying, Dingfeng Lv, Ping Zhang

**Affiliations:** ^a^ Department of Blood Transfusion, The First Affiliated Hospital of Ningbo University, Ningbo, Zhejiang, China; ^b^ Department of Hematology, The First Affiliated Hospital of Ningbo University, Ningbo, Zhejiang, China

**Keywords:** Hematopoietic stem cell transplantation, hematopoietic reconstitution, transfusion, risk factors, retrospective study

## Abstract

**Background:**

Hematopoietic stem cell transplantation (HSCT) cures many hematologic malignancies, but peri‑engraftment complications like delayed engraftment and high transfusion requirements remain challenging. Prior studies examined the independent effects of transplant type, hemoglobin, and platelets on post‑transplant outcomes, but whether their associations with these outcomes vary by transplant type is unknown.

**Methods:**

In this retrospective cohort of 341 HSCT patients, we examined whether the associations between pre-transplant hemoglobin and platelet levels and four key peri-engraftment outcomes (RBC transfusion volume, granulocyte engraftment time, megakaryocyte engraftment time, and platelet transfusion requirements) varied by transplant type (allogeneic vs. autologous). Multiple linear regression models for each outcome included diagnosis, transplant type,centered CD34+(C_CD34+),centered prothrombin time (C_PT), centered hemoglobin (C_Hb),and centered platelet (C_PLT) as main effects, with interaction terms: transplant type × C_Hb and transplant type × C_PLT. Bootstrap with 1000 resamples was used to estimate 95% CIs.

**Results:**

The transplant type × hemoglobin interaction was significant for granulocyte engraftment time (*p* = 0.014) and RBC transfusion volume (*p* = 0.029). Simple slope analyses showed that the adverse effect of allogeneic transplantation was largest at low hemoglobin (−1 SD: +2.67 days , +2.93 units) and smallest at high hemoglobin (+1 SD: +1.48 days , +1.43 units). No significant interactions were observed for platelet‑related outcomes (all *p* > 0.05).

**Conclusions:**

Higher pre‑transplant hemoglobin attenuated the adverse effects of allogeneic HSCT on granulocyte recovery and RBC transfusion requirements, suggesting baseline hemoglobin may serve as a useful risk indicator for peri‑engraftment management in allogeneic recipients, warranting prospective validation.

## 1. Introduction

Hematopoietic stem cell transplantation(HSCT) is a curative treatment for many malignant hematologic disorders. It is classified as autologous or allogeneic depending on the stem cell source. Autologous transplantation offers rapid immune reconstitution and eliminates the risk of graft‑versus‑host disease. In contrast, allogeneic transplantation effectively eradicates residual malignant cells *via* graft‑versus‑tumor effects, albeit with more complex immune complications[1–3]. Despite advances in transplantation, peri‑transplantation complications remain significant clinical challenges. Notably, the relative timing of post‑transplant granulocyte versus megakaryocyte engraftment influences risks of infection, bleeding, and hospitalization duration [4]. Additionally, peri‑engraftment red blood cell and platelet transfusions reflect resource utilization and transfusion‑related complication risks, respectively. Identifying predictors of these outcomes and their interactions is essential to optimize individualized management strategies.

The type of transplantation was the primary stratification variable for these outcomes. Compared with autologous recipients, allogeneic recipients had significantly longer time to granulocyte engraftment, a similar trend for megakaryocyte engraftment, and markedly higher rates of red blood cell and platelet transfusions [5,6]. Contributing factors included the more aggressive myeloablative conditioning typically used in allogeneic transplantation and the prolonged use of immunosuppressive medications [7]. Furthermore, baseline hemoglobin levels were negatively correlated with the need for red blood cell transfusions, while platelet levels predicted bleeding risk and transfusion thresholds [8,9]. Nevertheless, most previous studies focused on main effects, and few investigated the interaction between transplant type and hematologic markers. Consequently, direct evidence remains insufficient to determine whether identical hemoglobin or platelet levels have the same clinical significance across both transplant types.

Mechanistically, myeloablative conditioning creates a persistently hypoxic bone marrow microenvironment in recipients of allogeneic transplantation. This hypoxia activates the hypoxia‑inducible factor (HIF) pathway, which can inhibit the proliferation of hematopoietic stem cells [10,11]. Low hemoglobin levels further compromise tissue oxygenation, potentially exacerbating this hypoxic insult. The post‑transplant inflammatory environment, driven by conditioning‑induced tissue damage and subsequent graft‑versus‑host reactions, is characterized by elevated IFN‑γ and TNF‑α,which not only directly suppress hematopoietic progenitor differentiation but also synergistically stabilize HIF‑1α through the NF‑κB pathway [12,13]. Consequently, suppression of hematopoietic stem cells together with inflammatory cytokines sustains the inhibition of erythroid and granulocytic differentiation.Although granulocyte engraftment is primarily regulated by G-CSF, severe anemia is often a manifestation of underlying bone marrow suppression that involves impairment of multilineage hematopoietic reconstitution. In contrast, platelet engraftment is mainly governed by thrombopoietin and integrin signaling, and current evidence does not support a direct effect of the HIF pathway on megakaryocyte proliferation or maturation [14]. Moreover, megakaryocytes and their precursors appear relatively resistant to hypoxia‑induced cell cycle arrest, possibly due to constitutive expression of the anti‑apoptotic protein BCL‑XL [15]. These lineage‑specific differences in hypoxic sensitivity form the biological basis for our hypotheses.

Building upon this framework, the present study systematically investigated whether the associations between baseline hemoglobin and platelet levels and four key peri‑engraftment clinical outcomes, including red blood cell transfusion volume, time to granulocyte engraftment, time to megakaryocyte engraftment, and platelet transfusion requirements, varied by transplant type (allogeneic vs. autologous). Using multiple linear regression models with interaction terms (transplant type × hemoglobin and transplant type × platelets), we examined whether these pre-transplant hematological parameters differentially affect engraftment kinetics and transfusion needs across graft sources. By delineating these conditional relationships, this study provides a basis for a risk-stratification framework that may offer useful information for individualized blood product management and clinical decision-making in patients at risk of delayed hematopoietic recovery.

## 2. Materials and methods

### 2.1. Research design and subjects

**Figure 1.**
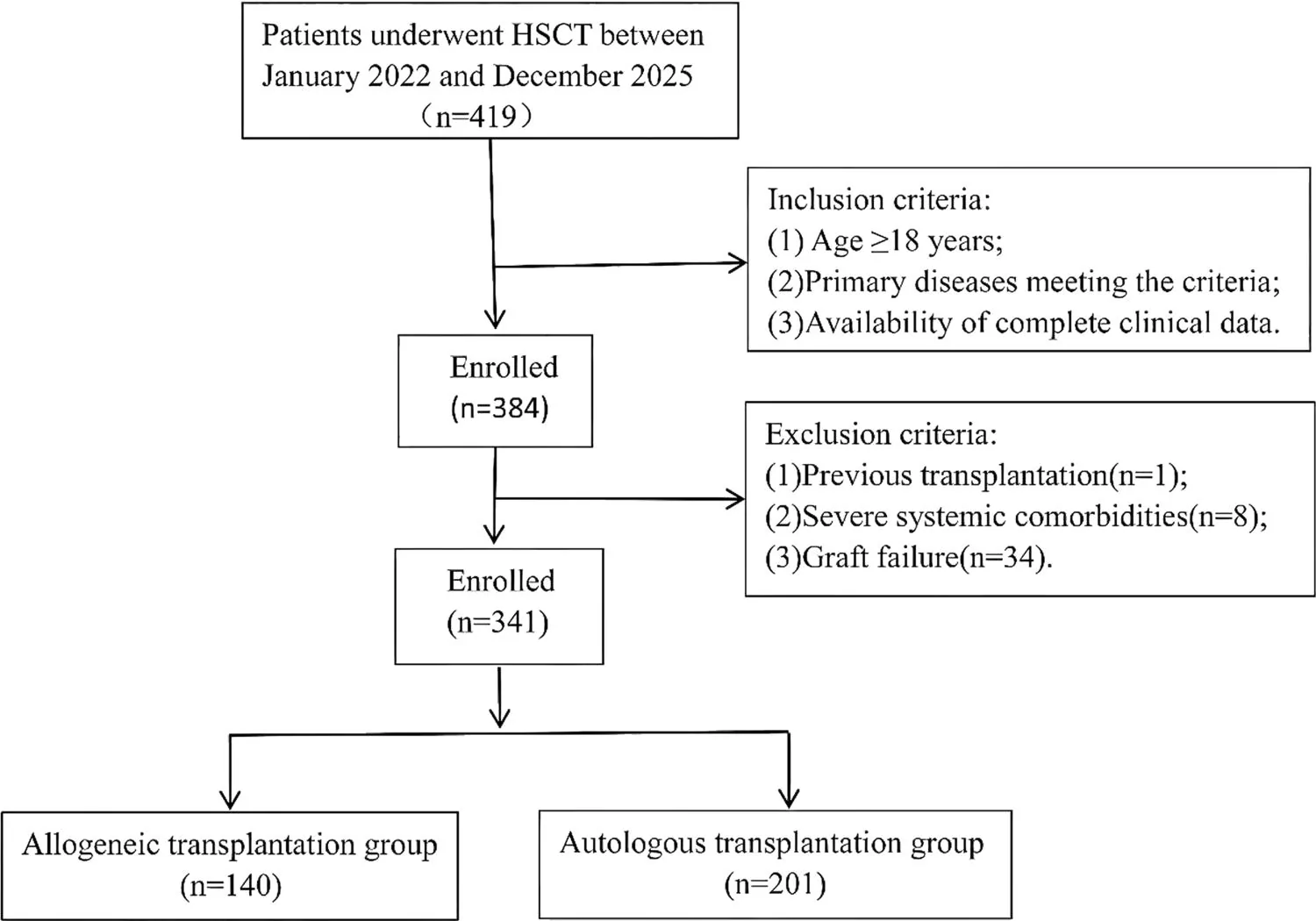
Inclusion flowchart of patient.

The First Affiliated Hospital of Ningbo University is a tertiary‑level hospital that houses a bone marrow transplant center equipped with 30 laminar flow beds and performs approximately 100 transplants annually. This retrospective cohort study included patients who underwent autologous or allogeneic hematopoietic stem cell transplantation at the hospital from January 2022 to December 2025, who were identified through the hospital’s electronic medical record system (Figure 1). Eligible patients were those whose primary diseases met the indications for hematopoietic stem cell transplantation and who had complete clinical data, including key pre‑ and post‑transplantation variables. Exclusion criteria were as follows: (1) a history of previous hematopoietic stem cell transplantation or solid organ transplantation; (2) presence of severe systemic comorbidities; (3) primary graft failure.

All patients included in or excluded from this study received treatment during the study period, and the study adhered to the ethical principles outlined in the Declaration of Helsinki (2013). The Ethics Committee of the First Affiliated Hospital of Ningbo University approved the study protocol(NO.2026-R154-01). The committee also granted a waiver for written informed consent due to the retrospective nature of the study.

### 2.2. Data collection

This study utilized data from the electronic medical record system and the blood transfusion management information system at the First Affiliated Hospital of Ningbo University. Trained researchers extracted and verified all data to ensure accuracy. The dataset included patient demographic characteristics, including age, gender, body mass index (BMI), blood type, primary disease diagnosis, remission status at transplantation and transplant type, as well as clinical parameters such as conditioning regimen, graft source, infection status during hospitalization, ABO compatibility (for allogeneic transplant recipients), and CD34+ cell count. Pré‑transplant laboratory parameters, comprising baseline values obtained within 24 h of admission, included prothrombin time (PT), activated partial thromboplastin time (APTT), alanine aminotransferase (ALT), aspartate aminotransferase (AST), albumin, blood urea nitrogen (BUN), serum creatinine (SCr), hemoglobin, and platelet count. Additionally, outcome measures encompassed time to megakaryocyte and granulocyte engraftment and volumes of red blood cell and platelet transfusions. All data were recorded using a pre-designed standard case report form.

### 2.3. Statistical analysis methods

We initially assessed normality of continuous variables using the Kolmogorov‑Smirnov test. Normally distributed variables were reported as mean ± standard deviation, with group comparisons using the independent samples t‑test. Non‑normally distributed variables were described as median (interquartile range), with group differences assessed using the Mann‑Whitney U test. Categorical variables were presented as frequencies (%), and group differences were analyzed using the chi‑square test. Correlations among continuous variables were examined using Spearman rank correlation. The four outcome variables were: (1) time to granulocyte engraftment (days); (2) time to megakaryocyte engraftment (days); (3) red blood cell transfusion (units); and (4) platelet transfusion (units). For each continuous outcome, we developed a multiple linear regression model that included independent variables selected based on univariate analysis (screening threshold *p* < 0.1) and clinical significance. To evaluate model stability, we performed bootstrap resampling with 1000 iterations for each regression model and calculated 95% confidence intervals (95% CI) for each regression coefficient. The primary objective was to determine whether the associations of hemoglobin and platelet levels with each outcome varied by transplant type. Based on the main‑effects model, we created two interaction terms: transplant type × hemoglobin and transplant type × platelets. Before constructing these interaction terms, we mean‑centered all continuous variables. We assessed the significance of each interaction term by adding it to the corresponding multiple linear regression model. For statistically significant interaction terms, we conducted simple slope analyses and plotted the interaction effects to illustrate the direction and magnitude of the moderating effect. All statistical analyses were performed using SPSS 27.0 (IBM Corp., Armonk, NY, USA), with statistical significance set at *p* < 0.05.

## 3. Results

### 3.1. Patient characteristics

A total of 341 HSCT patients were enrolled, including 140 allogeneic (41.06%) and 201 autologous (58.94%) patients. Baseline characteristics are compared in Table 1. Patients in the allogeneic group were younger than those in the autologous group (median 50.5 vs. 57.0 years, *p* < 0.001). The distribution of diagnoses differed significantly between the two groups (*p* < 0.001). Time to granulocyte engraftment and time to megakaryocyte engraftment were significantly longer in the allogeneic than in the autologous group (13 vs. 11 days and 13 vs. 12 days, respectively; both *p* < 0.001). In addition, red blood cell and platelet transfusions were significantly higher in the allogeneic group (4 vs. 0 units and 40 vs. 20 units, respectively; both *p* < 0.001). Results of normality testing for each variable are provided in (supplementary material Table S1). To further examine the impact of ABO compatibility on clinical outcomes, we performed subgroup analyses restricted to allogeneic transplant recipients stratified by ABO matching status. No significant differences were observed between ABO-compatible and ABO-incompatible patients across any of the outcomes examined. Detailed results are provided in (supplementary material Table S2).

**Table 1.** Baseline characteristics of patients underwent HSCT (*N* = 341).

Variable	Allogeneic transplantation group *n* = 140	Autologous transplantation group *n* = 201	Z/t/χ²	*p*-value
Age (years)	50.500(37.750,60.000)	57.000(51.000,63.000)	−4.724	<0.001**
PT (s)	11.550(11.000,12.500)	11.000(10.600,11.500)	5.157	<0.001**
ALT (U/L)	19.000(12.000,30.250)	17.000(12.000,24.000)	2.124	0.034*
AST (U/L)	19.000(16.000,26.000)	21.000(16.000,25.000)	−0.672	0.502
BUN (mmol/L)	4.705(3.908,5.905)	4.960(4.200,6.000)	−1.576	0.115
SCr (µmol/L)	63.000(53.000,72.000)	63.000(53.000,74.000)	−0.999	0.318
Hemoglobin (g/L)	92.000(70.000,109.000)	112.000(102.000,123.000)	−7.873	<0.001**
Platelet (×10⁹/L)	128.500(51.000,209.000)	189.000(140.000,231.000)	−5.496	<0.001**
CD34+(×10^6^/kg)	6.400(5.290,9.025)	7.320(4.880,10.300)	1.275	0.202
Platelet transfusion (U)	40.000(20.000,60.000)	20.000(20.000,30.000)	8.090	<0.001**
RBC transfusion (U)	4.000(2.000,6.000)	0.000(0.000,0.000)	10.285	<0.001**
Time to megakaryocyte engraftment (days)	13.000(12.000,16.250)	12.000(11.000,14.000)	5.538	<0.001**
Time to granulocyte engraftment (days)	13.000(11.000,15.000)	11.000(10.000,11.000)	10.316	<0.001**
BMI (kg/m²)	23.13 ± 3.32	23.62 ± 3.33	−1.341	0.181
APTT (s)	31.35 ± 3.40	31.08 ± 3.19	0.742	0.458
Albumin (g/L)	39.30 ± 4.07	39.33 ± 3.75	−0.075	0.940
Sex				
Female	56(40.00%)	96(47.76%)	1.710	0.191
Male	84(60.00%)	105(52.24%)		
Diagnosis				
ALL	28(20.00%)	3(1.49%)	225.829	0.000**
AML	45(32.14%)	15(7.46%)		
MDS	43(30.71%)	1(0.50%)		
MM	1(0.71%)	76(37.81%)		
NHL	5(3.57%)	94(46.77%)		
Others	18(12.86%)	12(5.97%)		
Blood type				
A	45(32.14%)	69(34.33%)	1.194	0.754
AB	7(5.00%)	15(7.46%)		
B	38(27.14%)	50(24.88%)		
O	50(35.71%)	67(33.33%)		
Remission status				
CR	140(100%)	201(100%)	–	–
Conditioning regimen				
MAC	131(93.57%)	201(100%)	10.888	0.001**
RIC	9(6.43%)	0(0%)		
Graft source				
Peripheral blood	139(99.29%)	201(100%)	0.033	0.856
Umbilical cord blood	1(0.71%)	0(0%)		
Infection status				
Positive	136(97.14%)	201(100%)	3.608	0.058
Negative	4(2.86%)	0(0%)		

*p < 0.05; **p < 0.01.

Note: CR: complete remission; MAC: myeloablative conditioning; RIC: reduced intensity conditioning.

### 3.2. Matrix of correlation coefficients between continuous variables

Based on univariate screening and clinical significance assessment, we included six variables in a multiple linear regression model: PT, hemoglobin, platelet,CD34+, diagnosis, and transplant type. Detailed results are provided in (supplementary material Table S3). To evaluate relationships between the continuous independent variables and the four continuous outcomes, and to assess potential multicollinearity, we performed spearman correlation analyses. Results are presented in Table 2; correlations involving the categorical variables (diagnosis and transplant type) and the outcomes are detailed in Table S3. No strong correlations were observed between any pair of variables (all |r| < 0.7), indicating a low risk of multicollinearity, which supports the construction of a stable and reliable multiple linear regression model.

**Table 2.** Results of correlation analysis between independent variables and outcome variables.

Variable	Mean	SD	1	2	3	4	5	6	7	8
1. CD34+(×10^6^/kg)	8.29	6.861	1							
2. PT(s)	11.433	1.306	0.030	1						
3. Hemoglobin (g/L)	103.323	23.159	−0.020	−0.268**	1					
4. Platelet (×10⁹/L)	177.956	103.012	0.162**	−0.140**	0.204**	1				
5. Platelet transfusion (U)	34.516	28.72	−0.188**	0.191**	−0.322**	−0.364**	1			
6. RBC transfusion (U)	2.183	3.519	−0.097	0.280**	−0.629**	−0.282**	0.547**	1		
7. Time to megakaryocyte engraftment (days)	13.598	5.299	−0.166**	0.136*	−0.228**	−0.278**	0.589**	0.405**	1	
8. Time to neutrophil engraftment (days)	11.71	2.38	−0.268**	0.241**	−0.327**	−0.306**	0.507**	0.490**	0.621**	1

*p < 0.05; **p < 0.01.

### 3.3. Results of multiple linear regression analysis for hematopoietic reconstitution outcomes

We built a multiple linear regression model to evaluate the impact of several factors on HSCT outcomes and to test the interaction between transplant type and both hemoglobin and platelet levels. The model included diagnosis, transplant type, mean‑centered prothrombin time (C_PT), mean‑centered hemoglobin (C_Hb), mean‑centered platelet count (C_PLT), and mean‑centered CD34+(C_CD34+),as well as the interaction terms transplant type × C_Hb and transplant type × C_PLT. We assessed the full model using variance inflation factor (VIF). VIF values for the main effects were all below 5, and those for the interaction terms were below 15. Moderately high VIFs for interaction terms are expected and acceptable in models with interaction effects and did not compromise the robustness of the coefficient estimates. We applied bootstrap resampling with 1000 iterations to estimate 95% confidence intervals; results are shown in Table 3. The interaction between transplant type and hemoglobin significantly influenced both time to granulocyte engraftment and red blood cell transfusion volume. To assess the robustness of our findings, we conducted a sensitivity analysis by additionally adjusting the primary regression models for conditioning regimen, graft source, and infection status. These variables were not included in the main models because of their limited variability across patients. As shown in (supplementary material Table S4), the direction, magnitude, and statistical significance of the interaction terms (transplant type × C_Hb and transplant type × C_PLT) remained virtually unchanged after these additional adjustments, confirming that our main results were not materially confounded by these factors. To further examine whether the observed interaction effects were consistent across different disease entities, we performed subgroup analyses stratified by diagnosis using multiple linear regression models to evaluate the significance of all interaction terms for each of the four outcomes within each disease subgroup. In these analyses, none of the interaction terms reached statistical significance in any of the disease-specific subgroups. Detailed results are provided in (supplementary material Table S5).

**Table 3.** Multiple linear regression models of factors on post-transplant hematopoietic recovery outcomes.

Variable	Time to megakaryocyte engraftment			Time to granulocyte engraftment			RBC transfusion			Platelet transfusion		
	B	*p*	Bootstrap 95%CI	B	*p*	Bootstrap 95%CI	B	*p*	Bootstrap 95%CI	B	*p*	Bootstrap 95%CI
Constant	10.407	0.001**	(7.914,12.980)	8.621	0.001**	(7.209,10.139)	−0.763	0.402	(−2.694,0.982)	26.311	0.002**	(13.376,39.280)
Diagnosis	0.019	0.931	(−0.410,0.432)	0.002	0.984	(−0.209,0.199)	−0.093	0.460	(−0.345,0.169)	−2.499	0.034*	(−4.574,-0.324)
Transplant type	2.074	0.005**	(0.992,3.098)	2.072	0.001**	(1.467,2.674)	2.178	0.001**	(1.370,3.047)	11.853	0.002**	(5.686,18.250)
C_CD34+	−0.015	0.709	(−0.113,0.038)	−0.030	0.049*	(−0.112,0.005)	−0.015	0.503	(−0.076,0.026)	−0.207	0.312	(−0.749,0.013)
C_PT	0.442	0.039*	(−0.070,1.261)	0.125	0.133	(−0.043,0.367)	0.225	0.059	(−0.009,0.642)	1.487	0.181	(−0.550,4.454)
C_Hb	0.008	0.854	(−0.047,0.066)	0.025	0.133	(−0.001,0.052)	0.001	0.964	(−0.048,0.043)	−0.035	0.877	(−0.378,0.287)
C_PLT	0.004	0.716	(−0.008,0.018)	0.002	0.557	(−0.005,0.010)	0.004	0.446	(−0.002,0.011)	0.005	0.925	(−0.050,0.079)
Transplant type × C_Hb	−0.024	0.364	(−0.072,0.021)	−0.026	0.014*	(−0.047,-0.005)	−0.032	0.029*	(−0.064,-0.002)	−0.073	0.600	(−0.339,0.202)
Transplant type × C_PLT	−0.007	0.259	(−0.017,0.002)	−0.003	0.217	(−0.008,0.002)	−0.005	0.126	(−0.010,0.001)	−0.036	0.238	(−0.092,0.006)
Model fit												
R²	0.159			0.365			0.412			0.223		
Adjusted R²	0.138			0.350			0.398			0.204		
F	F(8,332)=7.819,p = 0.001			F(8,332)=23.857,p = 0.001			F(8,332)=29.054,p = 0.001			F(8,332)=11.914,p = 0.001		

*p < 0.05; **p < 0.01.

### 3.4. Simple slope analysis of transplant type on outcomes at different hemoglobin levels

**Figure 2.**
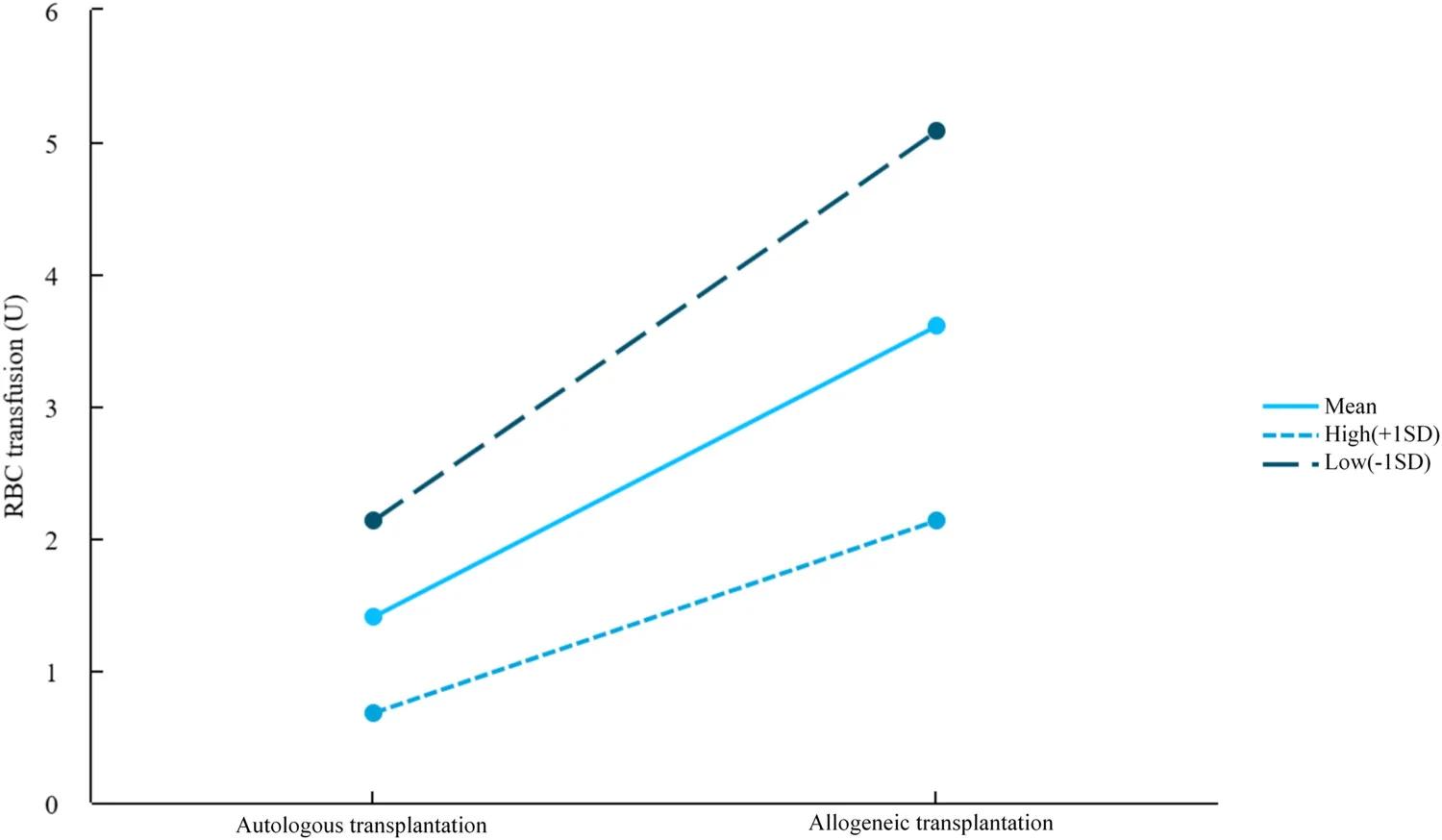
Predictive effect of transplant type on red blood cell transfusion at different hemoglobin levels.

**Figure 3.**
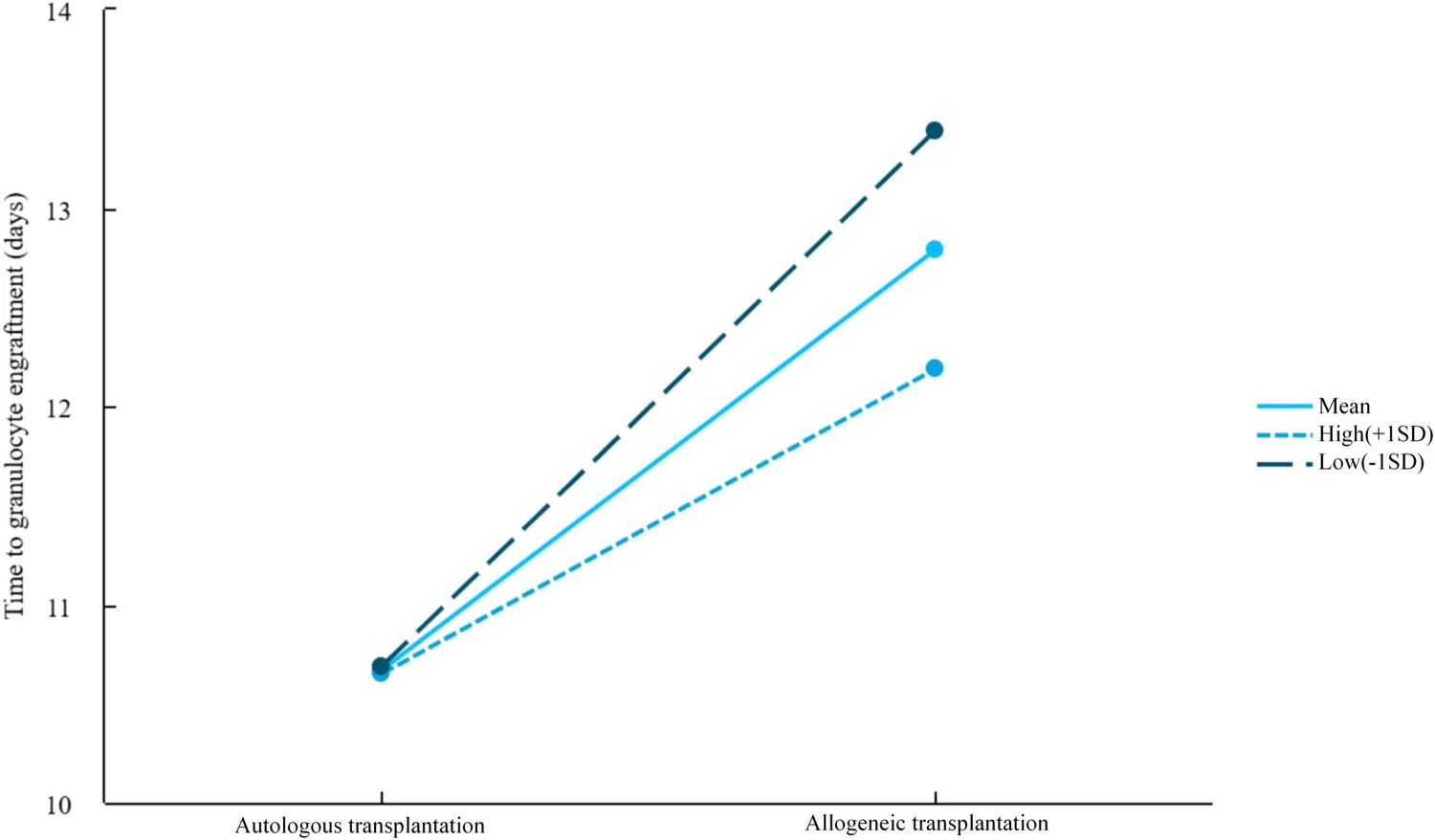
Predictive effect of transplant type on time to granulocyte engraftment at different hemoglobin levels.

For outcomes with significant interactions (i.e. time to granulocyte engraftment and red blood cell transfusion), we performed additional simple slope analyses stratified by transplant type to estimate effect sizes for allogeneic versus autologous (reference group) at different hemoglobin levels (mean and mean ± 1 SD). Results are presented in Table 4, and interaction effect plots are shown in Figures 2 and 3. The effect of allogeneic relative to autologous progressively diminished as hemoglobin levels decreased and increased as hemoglobin levels rose. Specifically, the effect was largest at low hemoglobin levels ( − 1 SD): granulocyte engraftment time + 2.668 days, red blood cell transfusion + 2.930 units; and smallest at high hemoglobin levels ( + 1 SD): + 1.476 days and + 1.427 units, respectively (all *p* < 0.01).

**Table 4.** Simple slope analysis of transplant type on granulocyte engraftment time and red blood cell transfusion at different hemoglobin levels.

Variable	C_hemoglobin level	Time to granulocyte engraftment			RBC transfusion		
		B	*p*	95%CI	B	*p*	95%CI
Transplantation type (Ref = Autologous)	–	–	–	–	–	–	–
Transplantation type = Allogeneic	Mean	2.072	0.001**	(1.511,2.633)	2.178	0.001**	(1.380,2.977)
	High(+1 SD)	1.476	0.001**	(0.717,2.235)	1.427	0.010*	(0.347,2.508)
	Low(-1 SD)	2.668	0.001**	(1.960,3.377)	2.930	0.001**	(1.921,3.938)

*p < 0.05; **p < 0.01.

## 4. Discussions

In this cohort of 341 HSCT recipients, we investigated whether the association between pre‑transplant hemoglobin and peri‑engraftment outcomes varies by transplant type. Our findings demonstrate that baseline hemoglobin shows a dose‑dependent and lineage‑specific predictive association with engraftment outcomes, which varies by transplant type. The association was significantly stronger in allogeneic than in autologous recipients for granulocyte engraftment and red blood cell transfusion, followed a dose‑response pattern that was most pronounced in patients with low hemoglobin and gradually diminished as hemoglobin levels increased, and was not observed for platelet‑related outcomes. Collectively, these findings suggest that hemoglobin level may serve as a transplant‑type‑specific risk indicator, with greater clinical utility in the allogeneic setting.

The differential association of hemoglobin with granulocyte engraftment and red blood cell transfusion between allogeneic and autologous recipients warrants careful consideration. Allogeneic recipients are known to experience delayed engraftment and higher transfusion requirements than autologous recipients [5,16]. However, prior studies have primarily reported main effects of transplant type, often adjusting for baseline hemoglobin as a covariate rather than examining whether its predictive value differs across transplant types. Our study extends this literature by demonstrating that the disparity between allogeneic and autologous recipients varies significantly as a function of hemoglobin level, with the interaction most pronounced at lower hemoglobin levels. Specifically, simple slope analyses revealed that the additional delay in granulocyte engraftment associated with allogeneic transplantation was 2.7 days at 1 SD below the mean hemoglobin level, compared with 1.5 days at 1 SD above the mean. Similarly, the incremental red blood cell transfusion requirement in allogeneic recipients ranged from 2.9 units at low hemoglobin to 1.4 units at high hemoglobin (Table 4). This differential association remained robust after we additionally adjusted for conditioning regimen, graft source, and infection status, as shown in Supplementary Table S4. These variables were not included in the primary models because of their limited variability in our cohort, which reflected the standardized protocols at our center. The stability of the interaction estimates across models with and without these covariates provides assurance that our main findings are not artifacts of confounding.

The lineage specificity of this association, which was present for granulocyte and erythroid outcomes but absent for megakaryocyte and platelet outcomes, deserves particular attention. This lineage specificity may be explained by the differential sensitivity of hematopoietic progenitors to microenvironmental stress. Erythroid and granulocytic progenitors appear more vulnerable to perturbations in oxygen delivery and inflammatory signaling, whereas megakaryopoiesis is primarily regulated by thrombopoietin and its receptor MPL, a pathway that may confer relative resistance to such stress [17,18]. This differential lineage sensitivity offers a plausible explanation for why we observed a significant hemoglobin‑dependent association for granulocyte and red blood cell outcomes but not for platelet‑related outcomes.

We also examined ABO compatibility in allogeneic recipients and found no significant association with any clinical outcome (Supplementary Table S2). This finding is consistent with a growing body of evidence suggesting that ABO incompatibility does not uniformly affect engraftment kinetics or platelet transfusion needs. A retrospective study of 76 allogeneic HSCT recipients reported that ABO incompatibility was not significantly associated with either neutrophil or platelet engraftment (*p* = 0.389 and *p* = 0.349, respectively) [19]. Similarly, a single‑center analysis of 715 allogeneic HSCT recipients found that ABO compatibility was not significantly associated with either neutrophil or platelet engraftment (*p* = 0.911 and *p* = 0.566, respectively) [20]. The absence of a significant ABO effect in our cohort may be attributable to the relatively small number of ABO‑incompatible allogeneic recipients, which limited our ability to detect modest differences. Alternatively, the standardized transfusion protocols at our center, including the use of blood components matched to both donor and recipient types as clinically indicated, may have mitigated the potential impact of ABO incompatibility on transfusion requirements.

The interpretation of the consistently null results for the interaction terms in disease‑specific subgroup analyses (Supplementary Table S5) requires careful consideration. The allogeneic and autologous groups differed substantially in underlying disease distribution, with acute leukemias predominating in the allogeneic cohort and plasma cell disorders or lymphomas in the autologous cohort. This disease‑related disparity inherently influences pre‑transplant hemoglobin levels, conditioning regimens, and supportive care needs, and we therefore adjusted for diagnosis as a categorical covariate in the regression models and performed diagnosis‑stratified subgroup analyses. One possible explanation for the null findings in these subgroups is that the association between hemoglobin and transplant‑type outcomes may not be uniform across all hematologic diseases. This could reflect genuine biological heterogeneity. Different disease entities and their prior treatments may affect hematopoietic reserve and bone marrow microenvironment in distinct ways. Alternatively, the lack of significant findings may reflect limited statistical power after stratification, as the reduced sample size within each diagnostic category diminished our ability to detect interaction effects of similar magnitude to those observed in the overall cohort. Both explanations are plausible, and distinguishing between them would require validation in larger, multicenter cohorts with sufficient power for disease‑specific analyses.

Our findings may have implications for peri‑engraftment management and supportive care in the transplant setting, particularly for allogeneic recipients. Specifically, low hemoglobin was associated with an additional 2.7 days of engraftment delay and an additional 2.9 units of red blood cell transfusion in allogeneic recipients. These differences may help clinicians identify high‑risk patients and inform more individualized supportive care planning. Given that delayed engraftment is a known risk factor for infectious complications and prolonged hospitalization [21,22], the hemoglobin‑associated differences in engraftment observed in our study may have implications for patient management and resource utilization. Red blood cell transfusion burden after allogeneic HSCT has been associated with increased non‑relapse mortality and decreased survival. In a cohort of 692 AML patients receiving allogeneic HSCT, failure to achieve RBC transfusion independence by day 30 was strongly associated with inferior 5‑year overall survival (39.2% vs. 59.6%; adjusted HR 1.83) and higher non‑relapse mortality (29.7% vs. 13.7%; HR 2.05) [23]. A separate retrospective analysis of 278 adult cord blood transplant recipients reported that RBC transfusion ≥18 units by day 30 was significantly associated with higher overall mortality (HR 1.86, *p* = 0.018) [24]. Together, these observations raise the question of whether the hemoglobin‑associated differences in transfusion requirements and engraftment kinetics identified in our cohort may be associated with longer‑term clinical outcomes beyond the peri‑engraftment period. Pre‑transplant hemoglobin level has been previously recognized as a useful marker for predicting post‑HSCT red blood cell requirements. Our findings extend this observation by demonstrating that this predictive value is amplified in the allogeneic setting.

Several limitations should be acknowledged. First, the single‑center retrospective design may limit the generalizability of our findings. Whether our results apply to centers with different treatment approaches or patient populations remains unknown. Second, although the overall sample size of 341 patients provided adequate power to detect the primary interaction effects, it was insufficient for robust subgroup analyses. Stratification by disease diagnosis led to small sample sizes in each subgroup, limiting our ability to detect interaction effects that might exist in specific disease entities or to make meaningful comparisons across diagnostic groups. Third, our analysis relied on a single baseline hemoglobin measurement obtained within 24 h of admission. We did not capture dynamic changes in hemoglobin during the peri‑transplant period, which could have provided additional predictive information. Fourth, we adjusted for key covariates and performed sensitivity analyses that included conditioning regimen, graft source, and infection status. However, residual confounding by unmeasured or imperfectly measured factors cannot be excluded. Finally, given the retrospective design and single‑center setting, our findings should be considered hypothesis‑generating. Prospective, multicenter studies with standardized protocols and sufficient statistical power are needed to validate these observations and to determine whether hemoglobin‑guided risk stratification translates into improved clinical outcomes.

## 5. Conclusions

In this cohort of 341 HSCT recipients, hemoglobin level was associated with the magnitude of the difference between allogeneic and autologous recipients in granulocyte engraftment time and red blood cell transfusion requirements. The difference was largest in patients with low hemoglobin and smallest in those with high hemoglobin. These findings suggest that baseline hemoglobin may be considered in risk stratification for peri-engraftment management, particularly in the allogeneic setting.

## Supplementary Material

Supplementary Material S4.docx

Supplementary Material S3.docx

Supplementary Material S1.docx

Supplementary Material S2.docx

Supplementary Material S5.docx

## Data Availability

The data sets used and analyzed during the current study are available from the corresponding author on reasonable request.

## References

[1] Bell B, Thursby S, Limbrick H, et al. A systematic review and metasynthesis of hematopoietic stem cell transplant (HSCT) patient’s experiences of long-term monitoring clinics from the patient’s perspective. J Patient Exp. 2024;11:23743735241229378. doi: 10.1177/23743735241229378. 38343689 PMC10854386

[2] Goldsmith SR, Ghobadi A, Dipersio JF, et al. Chimeric antigen receptor T cell therapy versus hematopoietic stem cell transplantation: an evolving perspective. Transplant Cell Ther. 2022;28(11):727–736. doi: 10.1016/j.jtct.2022.07.015. 35878743 PMC10487280

[3] Porrata LF. The impact of infused autograft absolute numbers of immune effector cells on survival post-autologous stem cell transplantation. Cells. 2022;11(14):2197. doi: 10.3390/cells11142197. 35883639 PMC9315986

[4] Zhong FL, He JJ, Bai KH, et al. Tigecycline-induced coagulation gene prognostic prediction model and intestinal flora signature in AML. Front Immunol. 2024;15:1486592. doi: 10.3389/fimmu.2024.1486592. 39611150 PMC11602473

[5] Ojha S, Patle V, Nagaraju P, et al. Blood components utilization in hematopoietic stem cell transplantation: thirteen-year analysis from an apex oncology center of India. Asian J Transfus Sci. 2023;17(2):221–228. doi: 10.4103/ajts.ajts_12_22. 38274961 PMC10807528

[6] Zhang R, Lv M, Yang D, et al. P1391: comparison of autologous and allogeneic stem cell transplantation for adult patients with Philadelphia chromosome-negative acute lymphoblastic leukemia in first complete remission. HemaSphere. 2022;6(S3):e76982a2–e7691276. doi: 10.1097/01.HS9.0000848424.55868.0c.

[7] Albalawi MAH, Al-Sayaghi KM, Hegazy SM, et al. Prophylactic fluoroquinolones in hematopoietic stem cell transplant recipients: a meta-analytic comparison of ciprofloxacin and levofloxacin. Medicine (Baltimore). 2025;104(19):e42317. doi: 10.1097/MD.0000000000042317. 40355203 PMC12074066

[8] Tabasi S, Parkhideh S, Roshandel E, et al. The association of disease type, pre-transplant hemoglobin level and platelet count with transfusion requirement after autologous hematopoietic stem cell transplantation. Caspian J Intern Med. 2021;12(4):544–550. doi: 10.22088/cjim.12.4.544. 34820061 PMC8590401

[9] Metcalf RA, Nahirniak S, Guyatt G, et al. Platelet transfusion: 2025 AABB and ICTMG international clinical practice guidelines. JAMA. 2025;334(7):606–617. doi: 10.1001/jama.2025.7529. 40440268

[10] Abbasizadeh N, Burns CS, Verrinder R, et al. Age and dose dependent changes to the bone and bone marrow microenvironment after cytotoxic conditioning with busulfan. Front Cell Dev Biol. 2024;12:1441381. doi: 10.3389/fcell.2024.1441381. 39139448 PMC11319712

[11] Li C, Wu B, Li Y, et al. Loss of sphingosine kinase 2 promotes the expansion of hematopoietic stem cells by improving their metabolic fitness. Blood. 2022;140(15):1686–1701. doi: 10.1182/blood.2022016112. 35881840

[12] Dou Y, Nian Z, Wang D, et al. Reconstituted CD74+ NK cells trigger chronic graft versus host disease after allogeneic bone marrow transplantation. J Autoimmun. 2024;147:103274. doi: 10.1016/j.jaut.2024.103274. 38936148

[13] Takahashi R, Takahashi H, Seoka L, et al. Interferon-γ-mediated selective inhibition of hematopoiesis and the clonal advantage of HLA-lacking hematopoietic stem progenitor cells in aplastic anemia. Leukemia. 2025;39(6):1490–1502. doi: 10.1038/s41375-025-02595-6. 40217071

[14] Wang W, Zhai J, Wang Y, et al. Integrin β3 dysregulation impairs megakaryopoiesis and microparticle production via disrupting Rho-associated protein kinase-dependent cytoskeletal dynamics. J Thromb Haemost. 2025;23(12):3965–3981. doi: 10.1016/j.jtha.2025.08.021. 40915574

[15] Josefsson EC, Vainchenker W, James C. Regulation of platelet production and life span: role of Bcl-xL and potential implications for human platelet diseases. Int J Mol Sci. 2020;21(20):7591. doi: 10.3390/ijms21207591. 33066573 PMC7589436

[16] Melik D, Aytan P, Gulum C, et al. Monocyte engraftment dynamics and flow cytometric analysis in allogeneic and autologous peripheral blood stem cell transplantation: a prospective study. BMC Cancer. 2026;26(1):766. doi: 10.1186/s12885-026-16115-x. 42067852 PMC13289530

[17] Du C, Chen J, Wang J. New insights into the generation and function of megakaryocytes in health and disease. Haematologica. 2025;110(7):1500–1512. doi: 10.3324/haematol.2024.287236. 40145277 PMC12208170

[18] Eliasson P, Rehn M, Hammar P, et al. Hypoxia mediates low cell-cycle activity and increases the proportion of long-term-reconstituting hematopoietic stem cells during in vitro culture. Exp Hematol. 2010;38(4):301–310.e2. doi: 10.1016/j.exphem.2010.01.005. 20138114

[19] Nalukettil BB, Biswas AK, Asthana B, et al. A retrospective study to assess the impact of ABO incompatibility on outcomes of allogeneic peripheral blood stem cell transplants at a tertiary care hospital in Western Maharashtra. Asian J Transfus Sci. 2023;17(2):202–209. doi: 10.4103/ajts.ajts_134_21. 38274976 PMC10807530

[20] Yu Y, Murphy MK, Tan V, et al. Impact of ABO compatibility on outcomes after allogeneic hematopoietic cell transplantation (HCT): increased risk of acute GVHD with ABO bidirectional mismatch, independent of traditional and emerging GVHD prophylaxis strategies. Cytotherapy. 2026;28(4):102005. doi: 10.1016/j.jcyt.2025.102005. 41719957

[21] Daqa AAM, Hamadi O, Daqqa AA, et al. Incidence and predictors of delayed hematologic engraftment after autologous stem cell transplantation and early post-transplant complications: a retrospective study. J Clin Oncol. 2026;44:e19103–e19103. doi: 10.1200/JCO.2026.44.16_suppl.e19103.

[22] Li N, Liang L, Xiang P, et al. Clonal haematopoiesis of indeterminate potential predicts delayed platelet engraftment after autologous stem cell transplantation for multiple myeloma. Br J Haematol. 2023;201(3):577–580. doi: 10.1111/bjh.18716. 36880443

[23] Yuan S, Yang D, Nakamura R, et al. Lack of RBC transfusion independence by Day 30 following allogeneic hematopoietic stemcell transplant strongly predicts inferior survival and high non-relapse mortality in acute myeloid leukemia patients. Transfusion. 2024;64(2):255–280. doi: 10.1111/trf.17714. 38225215

[24] Konuma T, Oiwa-Monna M, Mizusawa M, et al. Red blood cell transfusion burden by day 30 predicts mortality in adults after single-unit cord blood transplantation. Bone Marrow Transplant. 2019;54(11):1836–1846. doi: 10.1038/s41409-019-0555-8. 31089286

