# Supplementary material for: Association of hemoglobin level with granulocyte engraftment and red blood cell transfusion by transplant type after hematopoietic stem cell transplantation: a retrospective cohort study
Source: Ann Med. 2026 Sep 7;58(1):2729599. doi: 10.1080/07853890.2026.2729599 (PMC13552288; doi:10.1080/07853890.2026.2729599)
Supplement: Supplementary file 1 [file IANN_A_2729599_SM8324.docx]

Table S5.Disease-stratified sensitivity analysis of the interactions on outcomes

| Outcome | Variable | ALL | |  | AML | |  | MDS | |  | MM | |  | NHL | |  | Others | |
| --- | --- | --- | --- | --- | --- | --- | --- | --- | --- | --- | --- | --- | --- | --- | --- | --- | --- | --- |
|  |  | B | *p* |  | B | *p* |  | B | *p* |  | B | *p* |  | B | *p* |  | B | *p* |
| RBC transfusion | Constant | -0.799 | 0.952 |  | -1.408 | 0.450 |  | 1.533 | 0.884 |  | -0.635 | 0.604 |  | -1.903 | 0.213 |  | 0.325 | 0.928 |
|  | C_CD34+ | 0.087 | 0.613 |  | -0.075 | 0.293 |  | -0.264 | 0.328 |  | -0.006 | 0.592 |  | -0.025 | 0.361 |  | 0.137 | 0.467 |
|  | C_PLT | -0.012 | 0.791 |  | 0.001 | 0.952 |  | - | - |  | - | - |  | 0.005 | 0.521 |  | -0.035 | 0.457 |
|  | C_Hb | -0.253 | 0.838 |  | -0.133 | 0.260 |  | -0.050 | 0.107 |  | -0.017 | 0.010* |  | 0.011 | 0.866 |  | -0.182 | 0.279 |
|  | C_PT | 0.248 | 0.641 |  | -0.122 | 0.678 |  | 1.293 | 0.015* |  | 0.034 | 0.702 |  | 0.125 | 0.440 |  | 0.182 | 0.861 |
|  | Transplant type | 2.038 | 0.759 |  | 2.263 | 0.036* |  | 0.631 | 0.907 |  | 1.230 | 0.303 |  | 2.747 | 0.066 |  | 2.213 | 0.391 |
|  | Transplant type×C_Hb | 0.086 | 0.889 |  | 0.026 | 0.676 |  | - | - |  | - | - |  | -0.039 | 0.532 |  | 0.090 | 0.319 |
|  | Transplant type×C_PLT | 0.003 | 0.887 |  | -0.004 | 0.698 |  | -0.005 | 0.199 |  | -0.002 | 0.412 |  | -0.003 | 0.643 |  | 0.022 | 0.440 |
| Time to granulocyte engraftment | Constant | -1.009 | 0.895 |  | 8.402 | 0.001* |  | 6.662 | 0.273 |  | 8.709 | 0.001* |  | 9.647 | 0.001* |  | 10.665 | 0.001* |
|  | C_CD34+ | -0.117 | 0.238 |  | -0.119 | 0.020* |  | -0.053 | 0.732 |  | -0.001 | 0.948 |  | -0.059 | 0.018* |  | -0.100 | 0.425 |
|  | C_PLT | -0.030 | 0.253 |  | 0.024 | 0.044* |  | - | - |  | - | - |  | -0.003 | 0.685 |  | 0.037 | 0.231 |
|  | C_Hb | -0.943 | 0.194 |  | 0.012 | 0.883 |  | -0.043 | 0.017* |  | -0.006 | 0.451 |  | 0.064 | 0.278 |  | -0.120 | 0.280 |
|  | C_PT | 0.184 | 0.546 |  | -0.113 | 0.581 |  | 0.747 | 0.015* |  | -0.038 | 0.740 |  | 0.084 | 0.569 |  | 0.311 | 0.651 |
|  | Transplant type | 6.713 | 0.088 |  | 2.292 | 0.003* |  | 2.586 | 0.406 |  | 2.309 | 0.145 |  | 0.888 | 0.513 |  | 0.460 | 0.786 |
|  | Transplant type×C_Hb | 0.460 | 0.204 |  | -0.017 | 0.696 |  | - | - |  | - | - |  | -0.065 | 0.258 |  | 0.079 | 0.186 |
|  | Transplant type×C_PLT | 0.014 | 0.289 |  | -0.013 | 0.057 |  | -0.002 | 0.363 |  | -0.005 | 0.070 |  | 0.002 | 0.793 |  | -0.029 | 0.126 |

* p<0.05.

Note: For the MDS and MM subgroups, the interaction term could not be estimated due to perfect or near‑perfect multicollinearity between transplant type and diagnosis (tolerance < 0.001), as almost all MDS patients received allogeneic transplants and almost all MM patients received autologous transplants in our cohort.
