# Supplementary material for: Association of hemoglobin level with granulocyte engraftment and red blood cell transfusion by transplant type after hematopoietic stem cell transplantation: a retrospective cohort study
Source: Ann Med. 2026 Sep 7;58(1):2729599. doi: 10.1080/07853890.2026.2729599 (PMC13552288; doi:10.1080/07853890.2026.2729599)
Supplement: Supplementary file 2 [file IANN_A_2729599_SM8325.docx]

Table S2.Impact of ABO blood group matching on transfusion requirements and time to engraftment in allogeneic HSCT

| Variable | ABO-matched group  n=71 | ABO-mismatched group  n=69 | Z | *p*-value |
| --- | --- | --- | --- | --- |
| Platelet transfusion (U) | 40.000(30.000,50.000) | 40.000(20.000,60.000) | -0.346 | 0.728 |
| RBC transfusion (U) | 3.500(1.500,6.000) | 4.000(2.000,6.000) | 0.784 | 0.429 |
| Time to megakaryocyte engraftment (days) | 13.000(12.000,15.500) | 13.000(12.000,17.000) | 0.119 | 0.906 |
| Time to granulocyte engraftment (days) | 12.000(11.500,14.000) | 13.000(11.000,15.000) | 0.604 | 0.543 |

* p<0.05 ,** p<0.01
