# Supplementary material for: Association of hemoglobin level with granulocyte engraftment and red blood cell transfusion by transplant type after hematopoietic stem cell transplantation: a retrospective cohort study
Source: Ann Med. 2026 Sep 7;58(1):2729599. doi: 10.1080/07853890.2026.2729599 (PMC13552288; doi:10.1080/07853890.2026.2729599)
Supplement: Supplementary file 3 [file IANN_A_2729599_SM8326.docx]

Table S1. Baseline characteristics and normality test of the study population (N=341)

| Variable | Mean | SD | KS D | *p*-value |
| --- | --- | --- | --- | --- |
| Age (years) | 52.774 | 12.630 | 0.108 | 0.001** |
| BMI (kg/m²) | 23.418 | 3.332 | 0.060 | 0.170 |
| PT (s) | 11.433 | 1.306 | 0.141 | 0.001** |
| APTT (s) | 31.189 | 3.278 | 0.041 | 0.601 |
| ALT (U/L) | 24.311 | 31.944 | 0.267 | 0.001** |
| AST (U/L) | 24.364 | 18.989 | 0.246 | 0.001** |
| Albumin (g/L) | 39.321 | 3.878 | 0.044 | 0.499 |
| BUN (mmol/L) | 5.106 | 1.632 | 0.080 | 0.025* |
| SCr (µmol/L) | 65.921 | 25.277 | 0.155 | 0.001** |
| Hemoglobin (g/L) | 103.323 | 23.159 | 0.083 | 0.017* |
| Platelet (×10⁹/L) | 177.956 | 103.012 | 0.083 | 0.017* |
| CD34+(×10^6^/kg) | 8.290 | 6.861 | 0.189 | 0.001** |
| Platelet transfusion (U) | 34.516 | 28.720 | 0.231 | 0.001** |
| RBC transfusion (U) | 2.183 | 3.519 | 0.290 | 0.001** |
| Time to megakaryocyte engraftment (days) | 13.598 | 5.299 | 0.237 | 0.001** |
| Time to neutrophil engraftment (days) | 11.710 | 2.380 | 0.193 | 0.001** |

Note: KS D = Kolmogorov-Smirnov D statistic; **p* < 0.05; ***p* < 0.01.
