# Supplementary material for: Association of hemoglobin level with granulocyte engraftment and red blood cell transfusion by transplant type after hematopoietic stem cell transplantation: a retrospective cohort study
Source: Ann Med. 2026 Sep 7;58(1):2729599. doi: 10.1080/07853890.2026.2729599 (PMC13552288; doi:10.1080/07853890.2026.2729599)
Supplement: Supplementary file 4 [file IANN_A_2729599_SM8327.docx]

Table S3.Univariate analysis of variables associated with hematopoietic reconstitution outcomes

| Variable | Time to megakaryocyte engraftment | |  | Time to granulocyte engraftment | |  | RBC transfusion | |  | | Platelet transfusion | | |
| --- | --- | --- | --- | --- | --- | --- | --- | --- | --- | --- | --- | --- | --- |
|  | t/χ²/F | *p*-value |  | t/χ²/F | *p*-value |  | t/χ²/F | *p*-value |  | t/χ²/F | | *p*-value |  |
| Age (years) | -0.402 | 0.688 |  | -1.034 | 0.302 |  | -1.791 | 0.074 |  | -1.143 | | 0.254 |  |
| BMI (kg/m²) | -1.391 | 0.165 |  | -1.862 | 0.063 |  | -1.838 | 0.067 |  | -1.647 | | 0.101 |  |
| PT (s) | 3.766 | <0.001** |  | 4.285 | <0.001** |  | 4.775 | <0.001** |  | 3.4 | | <0.001** |  |
| APTT (s) | 1.353 | 0.177 |  | 0.79 | 0.430 |  | -0.057 | 0.955 |  | 0.897 | | 0.370 |  |
| ALT (U/L) | 0.126 | 0.900 |  | 1.923 | 0.055 |  | 1.133 | 0.258 |  | -0.296 | | 0.767 |  |
| AST (U/L) | -0.957 | 0.339 |  | 0.05 | 0.960 |  | 0.08 | 0.936 |  | -1.142 | | 0.254 |  |
| Albumin (g/L) | -1.296 | 0.196 |  | -2.939 | 0.004** |  | -2.991 | 0.003** |  | -0.146 | | 0.884 |  |
| BUN (mmol/L) | 3.051 | 0.002** |  | 1.925 | 0.055 |  | 1.504 | 0.134 |  | 2.35 | | 0.019* |  |
| SCr (µmol/L) | 1.261 | 0.208 |  | 0.233 | 0.816 |  | 0.846 | 0.398 |  | 0.953 | | 0.342 |  |
| Hemoglobin (g/L) | -5.207 | <0.001** |  | -7.508 | <0.001** |  | -11.112 | <0.001** |  | -5.774 | | <0.001** |  |
| Platelet (×10⁹/L) | -4.372 | <0.001** |  | -5.033 | <0.001** |  | -5.305 | <0.001** |  | -5.234 | | <0.001** |  |
| CD34+(×10^6^/kg) | -1.115 | 0.266 |  | -2.804 | 0.005** |  | -1.643 | 0.101 |  | -1.877 | | 0.061 |  |
| Transplantation type |  |  |  |  |  |  |  |  |  |  | |  |  |
| Autologous transplantation | 5.933 | <0.001** |  | 11.918 | <0.001** |  | 11.174 | <0.001** |  | 7.806 | | <0.001** |  |
| Allogeneic transplantation |  |  |  |  |  |  |  |  |  |  |  |  |  |
| Sex |  |  |  |  |  |  |  |  |  |  | |  |  |
| Female | 1.96 | 0.051 |  | 2.204 | 0.028 |  | 1.578 | 0.116 |  | 1.622 | | 0.106 |  |
| Male |  |  |  |  |  |  |  |  |  |  |  |  |  |
| Continued Table S3 | | | | | | | | | | | | |  |
| Variable | Time to megakaryocyte engraftment | |  | Time to granulocyte engraftment | |  | RBC transfusion | |  | | Platelet transfusion | | |
|  | t/χ²/F | *p*-value |  | t/χ²/F | *p*-value |  | t/χ²/F | *p*-value |  | t/χ²/F | | *p*-value |  |
| Diagnosis |  |  |  |  |  |  |  |  |  |  | |  |  |
| ALL | 7.348 | <0.001** |  | 17.696 | <0.001** |  | 24.517 | <0.001** |  | 15.321 | | <0.001** |  |
| AML |  |  |  |  |  |  |  |  |  |  |  |  |  |
| MDS |  |  |  |  |  |  |  |  |  |  |  |  |  |
| MM |  |  |  |  |  |  |  |  |  |  |  |  |  |
| NHL |  |  |  |  |  |  |  |  |  |  |  |  |  |
| Others |  |  |  |  |  |  |  |  |  |  |  |  |  |
| Blood type |  |  |  |  |  |  |  |  |  |  | |  |  |
| A | 0.766 | 0.514 |  | 2.578 | 0.054 |  | 1.104 | 0.348 |  | 1.221 | | 0.302 |  |
| AB |  |  |  |  |  |  |  |  |  |  |  |  |  |
| B |  |  |  |  |  |  |  |  |  |  |  |  |  |
| O |  |  |  |  |  |  |  |  |  |  |  |  |  |
| Remission status |  |  |  |  |  |  |  |  |  |  | |  |  |
| CR | - | - |  | - | - |  | - | - |  | - | | - |  |
| Conditioning regimen |  |  |  |  |  |  |  |  |  |  | |  |  |
| MAC | -0.358 | 0.721 |  | -1.940 | 0.053 |  | -1.234 | 0.218 |  | -1.051 | | 0.294 |  |
| RIC |  |  |  |  |  |  |  |  |  |  |  |  |  |
| Graft source |  |  |  |  |  |  |  |  |  |  | |  |  |
| Peripheral blood | 0.113 | 0.910 |  | -0.542 | 0.588 |  | 0.621 | 0.535 |  | 0.506 | | 0.613 |  |
| Umbilical cord blood |  |  |  |  |  |  |  |  |  |  |  |  |  |
| Infection status |  |  |  |  |  |  |  |  |  |  | |  |  |

Continued Table S3

| Variable | Time to megakaryocyte engraftment | |  | Time to granulocyte engraftment | |  | RBC transfusion | |  | | Platelet transfusion | | |
| --- | --- | --- | --- | --- | --- | --- | --- | --- | --- | --- | --- | --- | --- |
|  | t/χ²/F | *p*-value |  | t/χ²/F | *p*-value |  | t/χ²/F | *p*-value |  | t/χ²/F | | *p*-value |  |
| Positive | -0.152 | 0.879 |  | -0.456 | 0.649 |  | -0.181 | 0.857 |  | -0.209 | | 0.835 |  |
| Negative |  |  |  |  |  |  |  |  |  |  |  |  |  |

*p < 0.05; **p < 0.01.
