# Supplementary material for: Association of hemoglobin level with granulocyte engraftment and red blood cell transfusion by transplant type after hematopoietic stem cell transplantation: a retrospective cohort study
Source: Ann Med. 2026 Sep 7;58(1):2729599. doi: 10.1080/07853890.2026.2729599 (PMC13552288; doi:10.1080/07853890.2026.2729599)
Supplement: Supplementary file 5 [file IANN_A_2729599_SM8328.docx]

Table S4. Sensitivity analysis assessing the robustness of interaction effects to additional covariate adjustment

| Outcome | Variable | Sensitivity Analysis Model | | |
| --- | --- | --- | --- | --- |
|  |  | B | p | 95%CI |
| Time to granulocyte engraftment | Constant | 9.217 | 0.001** | (4.523,13.911) |
|  | Diagnosis | 0.001 | 0.999 | (-0.174,0.174) |
|  | Transplant type | 2.103 | 0.001** | (1.531,2.675) |
|  | C_CD34+ | -0.030 | 0.053 | (-0.060,0.001) |
|  | C_PT | 0.125 | 0.137 | (-0.040,0.289) |
|  | C_Hb | 0.025 | 0.136 | (-0.008,0.058) |
|  | C_PLT | 0.002 | 0.589 | (-0.005,0.010) |
|  | Graft source | 0.180 | 0.926 | (-3.654,4.014) |
|  | Infection status | -0.493 | 0.618 | (-2.434,1.449) |
|  | Conditioning regimen | -0.307 | 0.647 | (-1.622,1.008) |
|  | Transplant type×C_Hb | -0.026 | 0.014* | (-0.046,-0.005) |
|  | Transplant type×C_PLT | -0.003 | 0.218 | (-0.007,0.002) |
| RBC transfusion | Constant | 5.898 | 0.081 | (-0.740,12.536) |
|  | Diagnosis | -0.100 | 0.425 | (-0.346,0.146) |
|  | Transplant type | 2.314 | 0.001** | (1.504,3.123) |
|  | C_CD34+ | -0.012 | 0.577 | (-0.055,0.031) |
|  | C_PT | 0.228 | 0.054 | (-0.004,0.460) |
|  | C_Hb | 0.002 | 0.947 | (-0.045,0.048) |
|  | C_PLT | 0.004 | 0.464 | (-0.007,0.015) |
|  | Graft source | -4.289 | 0.121 | (-9.710,1.132) |
|  | Infection status | -1.248 | 0.372 | (-3.993,1.497) |
|  | Conditioning regimen | -1.228 | 0.195 | (-3.087,0.632) |
|  | Transplant type×C_Hb | -0.033 | 0.027* | (-0.062,-0.004) |
|  | Transplant type×C_PLT | -0.005 | 0.140 | (-0.011,0.002) |

*p < 0.05; **p < 0.01.

Note:Given that all patients had achieved complete remission (CR) before transplantation, remission status was not included in the regression analyses.Sensitivity analysis model additionally adjusted for conditioning regimen, graft source, and infection status.
